# Astrocytes and the Psychiatric Sequelae of COVID-19: What We Learned from the Pandemic

**DOI:** 10.1007/s11064-022-03709-7

**Published:** 2022-08-03

**Authors:** Luca Steardo, Luca Steardo, Caterina Scuderi

**Affiliations:** 1 Psychiatric Unit, Department of Health Sciences, University Magna Graecia of Catanzaro, Catanzaro, Italy; 2 Department of Physiology and Pharmacology “Vittorio Erspamer”, SAPIENZA University of Rome, Rome, Italy; 3 Università Giustino Fortunato, Benevento, Italy

**Keywords:** Astrocyte, SARS-CoV-2, COVID-19, Neuroinflammation, Reactive gliosis, Neuropsychiatric disorders

## Abstract

COVID-19, initially regarded as specific lung disease, exhibits an extremely broad spectrum of symptoms. Extrapulmonary manifestations of the disease also include important neuropsychiatric symptoms with atypical characteristics. Are these disturbances linked to stress accompanying every systemic infection, or are due to specific neurobiological changes associated with COVID-19? Evidence accumulated so far indicates that the pathophysiology of COVID-19 is characterized by systemic inflammation, hypoxia resulting from respiratory failure, and neuroinflammation (either due to viral neurotropism or in response to cytokine storm), all affecting the brain. It is reasonable to hypothesize that all these events may initiate or worsen psychiatric and cognitive disorders. Damage to the brain triggers a specific type of reactive response mounted by neuroglia cells, in particular by astrocytes which are the homeostatic cell par excellence. Astrocytes undergo complex morphological, biochemical, and functional remodeling aimed at mobilizing the regenerative potential of the central nervous system. If the brain is not directly damaged, resolution of systemic pathology usually results in restoration of the physiological homeostatic status of neuroglial cells. The completeness and dynamics of this process in pathological conditions remain largely unknown. In a subset of patients, glial cells could fail to recover after infection thus promoting the onset and progression of COVID-19-related neuropsychiatric diseases. There is evidence from post-mortem examinations of the brains of COVID-19 patients of alterations in both astrocytes and microglia. In conclusion, COVID-19 activates a huge reactive response of glial cells, that physiologically act as the main controller of the inflammatory, protective and regenerative events. However, in some patients the restoration of glial physiological state does not occur, thus compromising glial function and ultimately resulting in homeostatic failure underlying a set of specific neuropsychiatric symptoms related to COVID-19.

## The Role of Astrocytes in the Brain: May the Homeostatic Cells Par Excellence Become SARS CoV-2 Damage Effectors?

Astroglia control such a huge number of functions that they can be considered to take part in any circumstance in which there is a disturbance of cerebral homeostasis. In fact, following any brain insults, these cells become reactive by profoundly modifying their morphology and functions. This complex response is part of the homeostatic tasks that glial cells perform physiologically and has as its objective the containment of the damage and the return to a homeostatic condition. However, glia changes are not always restored in a timely manner thus causing brain damage. At the beginning of 2021, we hypothesized that the neuropsychiatric consequences of COVID-19 are maladaptive glial recovery to blame [1, 2]. This hypothesis is now reinforced by growing evidence which is summarized in the following paragraphs.

Glial cells are heterogeneous neural cells exerting a plethora of functions mainly aimed at preserving the central nervous system (CNS) homeostasis. Glial cells are usually classified into microglia and macroglia. This latter includes astrocytes, oligodendrocytes, and oligodendrocyte precursors, also known as NG-2 glia or synantocytes [3, 4]. Microglia are of myeloid origin; their precursors migrate into the neural tube early in development. Microglial cells undergo profound metamorphosis acquiring specific 'neural'-like morphology and expressing numerous receptors for neurotransmitters and neurohormones [5]. Microglial cells contribute to CNS physiology and are mounting CNS defence in pathology being the main immunocompetent cells of the nervous tissue. Microglia scan the tissue and modify their morphology and functions if and when necessary [6]. Microglia are crucial for the formation, shaping, and functioning of synapses [7, 8], fundamental for brain development during pre- and post-natal periods. Erratic execution of synaptic elimination by microglia during early post-natal life is associated with anomalous functional connectivity, hippocampal long-term potentiation impairment, and aberrant behaviours [9, 10]. Microglia activate phagocytosis to incorporate waste products, cellular debris, and pathogens. They could react to pro-inflammatory stimuli by releasing cytokines, chemokines, and reactive oxygen and nitrogen species [11].

Oligodendrocytes are macroglial cells chiefly responsible for the formation of the myelin sheath around axons, thus being a fundamental element of the brain connectome [12]. Oligodendrocytes also support axons through cytoplasmic-rich myelinic channels that allow bidirectional movement of macromolecules under the myelin sheath [12–14]. Oligodendrocytes originate from precursor cells mainly localized in the ventricular zones of the brain, from which they migrate to colonise the developing CNS and became mature cells. This process starts shortly before birth and continues lifelong. The maturation of oligodendrocytes is usually accelerated in case of CSN injury, aging, or brain diseases, in order to replace the lost myelin [15]. Functions of oligodendrocyte precursors, also known as NG-2 glia, remain to be fully characterised. These cells express several receptors and ion channels, their receive synaptic contacts and may contribute to homeostatic control of the nervous tissue. Since NG-2 glia can differentiate into oligodendrocytes, they play a role in myelination and brain plasticity [16, 17].

Astroglia are fundamental for the maintenance of CNS homeostasis at molecular, cellular, organ, and system levels of organization [18, 19]. Several morphologically and functionally distinct subtypes of astroglial cells have been identified (e.g., protoplasmic and fibrous astrocytes of the grey matter, velate astrocytes of the cerebellum and olfactory bulb, radial astrocytes, perivascular and marginal astrocytes, ependymocytes, and many others) [19]. Astrocytes form the parenchymal part of the blood–brain barrier (BBB), which controls the exchanges of molecules and fluids between the brain and the periphery as well as restricts pathogens and cells brain invasion [18, 20–22]. Astrocytes regulate interstitial pH, control the concentration of extracellular ions, and scavenge reactive oxygen species [19]. Astrocytes are the central part of neuro-vascular unit, and they are involved in the regulation of local hyperaemia through the release of vasoactive molecules [23, 24]. Astrocytes are a part of the gliocrine system secreting about 200 molecules, including neurotrophic and synaptogenic factors as well as providing energy support to other neural cells [25]. Finally, astrocytes are fundamental components of the glymphatic system responsible for cleansing the nervous tissue [26]. Through morphological contact with synapses, astrocytes form the 'synaptic cradle', regulating all aspects of synaptic functions from synaptogenesis to synaptic maintenance and extinction [27]. In particular, astrocytes are indispensable for the control of neurotransmitter homeostasis in the brain [18, 28]. Astrocytes control so many cerebral functions that they are considered the homeostatic cells par excellence. As a consequence, any changes in the physiological performance of astrocytes may have a role in the etiology or progression of neurological pathologies.

Any insult to the CNS, including invasion of pathogens, triggers glial response, known as reactive astrogliosis [29]. Reactive glia play a fundamental defensive role, starting a series of responses aimed at restoring the lost homeostasis. Peripheral proinflammatory cytokines may induce microgliosis and astrogliosis whenever a CNS insult occurs, including viral infections [30]. Whenever glial cells lose their homeostatic activities, neuronal cells suffer. If the alteration of glial cells persists the irreversible damage to the nervous tissue may occur [3, 31–34].

## The Neurotropism of SARS-CoV-2 and the Neurological Manifestations of COVID-19

SARS-CoV-2 which causes COVID-19 emerged from China in 2019. The virus spread rapidly through the world triggering the pandemic. SARS-CoV-2 is a positive-sense single-stranded β-coronavirus belonging to the family of Coronaviridae, the largest group of viruses causing respiratory and gastrointestinal infections [35, 36]. Historically, coronaviruses received little attention due to their scant effects on humans. This changed in 2002 when atypical pneumonia spread from the Guangdong province to more than twenty countries. This illness was named severe acute respiratory syndrome (SARS) and the identified etiological β-coronavirus was named SARS-CoV [37]. Although COVID-19 seems to have a lower case-fatality rate than SARS (about 2.3% versus about 6.4%, respectively [38]), the massive spread of the infection has claimed over 6.3 million casualties worldwide (WHO web-dashboard data updated on June 29th, 2022). The majority of the infected people appear to have eliminated the coronavirus from their bodies after a few weeks and resume normal activity. However, about the 40% of infected people experience a variety of symptoms (loss of smell and/or taste, fatigue, cough, aching pain, "brain fog," insomnia, shortness of breath, and tachycardia) after several weeks and are diagnosed with the so-called long COVID syndrome.

To invade cells the SARS-CoV-2 spike protein binds to the angiotensin-converting enzyme 2 (ACE2) receptor which undergoes proteolytic processing by the transmembrane protease serine 2 [39–41]. In addition, both basigin (also known as CD147) and neuropilin-1 were identified as docking receptors for the SARS-CoV-2 virus [42–44]. After the first signs of the illness, the patient experiences a short recovery time, in which symptoms attenuate, usually followed by a more severe symptomatic. The human immune response induced by SARS-CoV-2 should develop in two phases. The constitutive adaptive immune response is activated at the beginning of the disease fighting the virus that actively replicates to colonise and damage the cells of the affected tissues, mostly lungs [38, 45]. A second phase, that take place in severe cases of COVID-19, defined as a severe acute respiratory distress syndrome (ARDS), is characterised by the so-called “cytokine storm” that is due to the hyperactivation of the immune system, accompanied by a massive release of proinflammatory mediators, cytokines, and chemokines [46, 47]. The hyperactive immune response impacts upon many organs and systems, underlying the multi-organ pathology observed in COVID-19 patients [48]. Thus, the cytokine load has also become the major hallmark in COVID-19 patients [49]. Growing clinical data suggest that patients having pre-existing conditions, such as obesity, cardiovascular diseases, hypertension, dyslipidemia, have a higher risk of developing severe or fatal COVID-19 [50–52].

Extrapulmonary manifestations of COVID-19 including neurological symptomatology, primarily anosmia and ageusia, are frequently reported [53–55]. Neurological symptoms in COVID-19 patients are grossly underestimated, especially because many severely ill patients are sedated and on ventilators [56]. However, cases of encephalitis, strokes, confusion, seizures, and brain inflammation have been reported [57–59]. A retrospective clinical study has provided evidence for substantial incidence of neurological and psychiatric events in patients during the first 6 months after getting COVID-19. The risk for neurological and psychiatric sequelae seems to be greatest in patients who had severe COVID-19 [60]. Cognitive deficits and depression have been seen in patients that recovered from mild COVID-19 [61].

The capability of SARS-CoV-2 to enter the CNS has been suggested by analogy with the neurotropism of other members of group 2 of the β-coronavirus family [62–67], to which SARS-CoV-2 belongs. Among several suggested routes of entry, the most studied and acknowledged is binding to ACE2 which is expressed in the CNS, mostly by endothelial cells [68] but also by both neurones and glial cells [69–71]. SARS-CoV-2 engages ACE2 as the entry receptor and employs the cellular serine protease TMPRSS2 for spike protein S cleavage [41]. This activates virus endocytosis controlled by endosomal proton pump and NAADP-sensitive intracellular two-pore channel 2 [72]. The ACE2 is expressed in the brain stem [69, 70], populating highly vascularised brain structures lacking the BBB like the circumventricular organs, the nucleus of the tractus solitarius, paraventricular nucleus, and rostral ventrolateral medulla [73]. Such distribution makes these regions more vulnerable to peripheral neurotoxic molecules or invasive agents, like SARS-CoV-2.

Another proposed route for viral entry to the brain is the invasion and consequent lesion of the olfactory system, which is consistent with the clinical data reporting that infection with SARS-CoV-2 is associated with high rates of disturbances in smell and taste perception, including anosmia [74–78]. Recently the cell types in the olfactory epithelium and olfactory bulb that express the SARS-CoV-2 cell entry molecules have been identified. Single-cell sequencing revealed that ACE2 is expressed in support cells, stem cells, and perivascular cells, rather than in neurons [79]. Through the olfactory system, the virus could spread into the brain stem, possibly compromising the respiratory centres [80]. Magnetic resonance imaging (MRI) investigations seem to corroborate that virus may enter the brain through the trans-nasal route [81–84], however, further studies are needed to better define their neuroradiologic interpretation.

Alternatively, the SARS-CoV-2 could penetrate through the median eminence, whose capillaries and tanycytes are thought to express ACE2, reaching the hypothalamus [85], and from there it could spread to the entire brain*.*

Brain infiltration of immune cells carrying the virus (a viral reservoir [86]) may represent another route of the virus entry. Vessels, meninges, and the choroid plexus have been proposed as entry points for infected monocytes, neutrophils, and T cells. However, conclusive evidence of infection through these routes has yet to be provided [87]. As suggested by some authors, some neurological symptoms and damage are the result of the body’s own immune system overreacting after encountering the virus. Some subjects inadvertently make ‘autoantibodies’ that attack their own tissue [88]. These autoantibodies can pass via the BBB, and contribute to both short- and long-term conditions, including neurological disorders ranging from brain fog to psychosis.

Evidence has also accumulated that the virus SARS-CoV-2 can affect the brain by reducing blood flow to it. In this way, SARS-CoV-2 infection impairs neurons function and ultimately killing them. Lastly, a leaky or dysfunctional BBB could facilitate the entry of the virus, as in other kinds of infections. For instance, the human immunodeficiency virus (HIV)-1 downregulates the expression of tight junction proteins, compromising BBB integrity [89]. Numerous studies highlight that systemic inflammation could damage glia limitans and damage the BBB [90]. Thus, the hyperreactive immune response triggered by SARS-CoV-2 may compromise the integrity of the BBB. Severe COVID-19 is often associated with comorbidities, such as CNS hypoxia due to respiratory failure, thrombotic microangiopathy, or pre-existing neurological diseases, which all may increase BBB permeability facilitating the entry of the virus into the brain [91]. The reported presence of SARS-CoV-2 in patients cerebrospinal fluid (CSF) and brain tissue suggests that once in the body the virus can reach the brain [92–94]. This is also true for SARS-CoV-1 [65, 66].Finally, the observation in post-mortem brain tissues of SARS-CoV-2 signal not coinciding with immune cell infiltration suggests that virus-related neurological complications could be the direct consequence of the neurotropism of SARS-CoV-2 [95, 96].

## The astrocyte response to viruses, including SARS-CoV-2

Typically, whenever any virus enters the CNS, the innate immune response activates. Both immune and neural cells participate in this process, cooperating in removing the pathogen. Astrocytes control the communication between resident and infiltrating immune cells and regulate the effector functions of antiviral T and B cells in the CNS compartments [97, 98]. Astrocytes respond quickly to brain insults, viruses including, by virtue of their functions of monitoring and preserving the brain homeostasis. In response to brain insults, astrocytes initiate the programme of reactive astrogliosis generally characterised by increased levels of the intermediate filament proteins glial fibrillary acidic protein (GFAP), vimentin, and nestin, as well as by hypertrophy of astrocytic processes, although in some cases atrophy has been documented too. In specific conditions, such as acute trauma, astrocytes may proliferate, regulate scar formation by fibroblasts and form new barriers around lesioned foci [29, 31, 99]. Reactive astrocytes are generally neuroprotective because they amplify homoeostatic cascades, detect and remove toxic substances and promote regeneration. At the same time, during viral infections, astrocytes and microglia may also become long-term virus reservoirs in the absence of efficient innate immune-mediated clearing mechanisms [100]. Viruses-induced rise in interleukin(IL)-1β and tumor necrosis factor(TNF)-α may cause changes in the metabolic phenotype of astrocytes, resulting in reduced glycogen storage and lactate transport, fundamental for energy support for neurons [97, 101]. In HIV-1 infection, astroglia release cytokines and chemokines able to reduce viral replication. Concurrently, when HIV infects astrocytes, it impairs their functions by forcing them to produce viral proteins, thus causing neuronal damage [102–105]. Furthermore, HIV-1 infected astrocytes release membrane HIV-1 Tat protein triggering mitochondrial dysfunction and neuronal death [106]. Proinflammatory cytokines secreted by microglial cells may promote astrogliosis whenever a CNS insult occurs, including viral infections [30, 107]. Astrogliosis and microgliosis could lead both of the cell types to gain aberrant functions or lose fundamental ones, resulting in neuronal damage [29, 108].

Murine coronavirus, MHV-A59, could infect the brain and its CNS effects were mediated by the cytokine release from reactive microglia and astrocytes. The authors documented that the cytokines released from both cell types were complementary, resulting in elevated levels of IL-1β, IL-6, interferon(INF)s, and TNF-α. Of note, they did not detect the release of the anti-inflammatory cytokines IL-4 and IL-10 [109]. In a SARS-CoV-1 patient, necrosis of neurons, broad hyperplasia of glial cells, and encephalic oedema have been reported. High plasma level of the chemokine Mig, a monokine induced by the INF-γ, that promotes the host defence by attracting activated T cells, natural killer (NK) cells, and CXCR3 expressing monocytes [66] has also been detected. Several studies indicated that SARS-CoV-2 affects astrocytes. A recent post-mortem investigation demonstrated that astrocytes are the main sites of viral infection within the CNS and that SARS-CoV-2-infected cells exhibit marked metabolic changes [110]. These authors suggest that astrocyte functions are impaired since they detected a reduction of the metabolites used to fuel neurons and produce neurotransmitters. In cortical tissue cultures and cortical organoids exposed to SARS-CoV-2, it has recently been demonstrated significant infection and viral replication in astrocytes, but minimal infection in other cell types [111].The same group reported that infected astrocytes had a corresponding increase in reactivity characteristics, growth factor signaling, and cellular stress. Signs of astrocyte reactivity have long been proposed in COVID-19 patients. For instance, elevated GFAP was found in the white matter of a COVID-19 patient, with encephalomyelitis-like brain damage, oligodendrocytic apoptosis and axonal injuries [112]. Plasma levels of both GFAP and neurofilament light chain protein (NfL), a biomarker predictive of intra-axonal neuronal injury, were measured in 47 patients with mild, moderate, or severe COVID-19 and matched controls. GFAP was found elevated in moderate/severe stages of the disease. This suggests that astrogliosis could be an early response after SARS-CoV-2 infection of the CNS [113]. In COVID-19-related acute necrotising encephalopathy virus was detected in the CSF, together with extremely high levels of NfL and GFAP, 19 days after the onset of the symptoms and even after testing negative twice [94]. These clinical studies indicate that astrocytes could be in a reactive state in COVID-19 patients. Consistently, the damage of the BBB and the strong lymphopenia observed during COVID-19 could promote the persistence of the virus, thus sustaining neuroinflammation and reactive gliosis. The resulting brain tissue alteration could explain some of the clinical features observed in COVID-19 patients who, despite overcome pneumonia, present cognitive impairment associated with behavioural changes [2, 114–117].

## Neuropsychiatric Consequences of COVID-19

CNS viral infections induce cognitive, mood, and motor deficits that may persist beyond the acute phase of the disease. In many cases, CNS sequelae may be provoked by irreversible damage to both neurons and glia triggered by pathogens [118]. Otherwise, infection-driven neuroinflammation can disturb brain homeostasis and circuit functioning inducing long-term deficits resulting in behavior alterations [119]. In this context, focusing on the neuropsychiatric sequelae that emerged following the SARS -COV 2 infection, longitudinal epidemiology research has revealed a broad spectrum of long-term consequences in patients who survived to COVID-19 pandemic, providing evidence that almost 80% of subjects discharged from hospital complained at least one of the following symptoms including fatigue, muscle weakness, myalgia, dizziness, headache sleep disturbances, brain fog, cognitive impairment, depression or anxiety in addition to cardiopulmonary manifestations [120–122]. Inevitably, the frequent persistence of this condition up to six months and beyond together with the failure of any effective treatment has a considerable impact on the quality of life of the affected subjects, keeping them out of work and social life [123]. Evidence that COVID-19 is followed by a significant rate of neuropsychiatric diagnoses over the subsequent six months has been further confirmed by a robust retrospective cohort study [117]. On the basis of this data, particular interest was drawn from the persistence of neuropsychiatric symptoms in convalescent patients or from their late appearance in subjects completely restored by the viral infection [60, 124, 125]. This should not be surprising, as similar features with significant neurological and mental complains were already reported in acute or post-disease phases during other previous coronavirus outbreaks. The experience gained with neurological and psychiatric manifestations of MERS and SARS would provide the right framework for better exploring CNS complications occurring during SARS-CoV-2 infection [126]. Post-COVID-19 psychiatric pathology frequently begins with a fatiguing feeling of asthenia, with a sense of apathy resulting in a condition of reduced interest in interpersonal relationships, and a decreased pleasure in carrying out those occupations that were previously a source of satisfaction. The appearance of sleep–wake rhythm disturbances and a progressive decrease in mood testify to the onset of an overt depression [127]. Depression following pandemics is considered one of the most significant public health concerns. A recent study reported a long-term prevalence beyond twelve months of 18.3% [120], while another investigation on the same topic suggested an overall depression prevalence of 27.9% [128]. According to some authors, the depression observed in the condition defined as long COVID appears to be characterized by manifestations that distinguish it from the canonical major depressive disorder [119]. Highlights of post-COVID-19 depression include a higher incidence of psychotic traits, marked motor agitation, evident neurocognitive deterioration, and profound changes in the sleep–wake rhythm. Psychotic anomalies, consisting in delusions, hallucinations, thoughts, disorganized may initially appear at the height of the COVID-19 pathology, and may also persist when the delirium is over and the infection is resolved. Psychotic manifestations many times emerge weeks or months after healing from the infection, not accompanied by delirium or confusion, mimicking the onset of a primary psychotic episode. Sleep disorders frequently complicate the clinical picture of long COVID-19 and are characterized by marked difficulty in initiating sleep rather than keeping it uninterrupted [129]. These sleep disturbances frequently occur in the younger population experiencing COVID-19 if even asymptomatic, without significant anxiety levels about the outcome and consequences of the infection. Sometimes insomnia remains even beyond the disappearance of the other disorders that had characterized the long COVID-19, in the absence of a manifest anxiety and an overt decline in mood, therefore leaving insomnia without a clear explanation [2, 129, 130]. A recent report by Jahrami et al. assessing the impact of the COVID-19 pandemic on the prevalence and severity of sleep problems among patients with COVID-19 indicated a high frequency of disturbed sleep, with an average rate of 74.8% [131]. Such an important incidence of sleep disturbances in patients who recovered from the acute SARS-CoV-2 infection could be explained by the interaction between sleep impairment and immune system dysfunction [132]. In fact, sleep and the immune system, according to researchers, interact bidirectionally. This hypothesis is supported by altered sleep patterns during viral infections with the release of inflammatory molecules, particularly in the acute phase of the immune response and the development of recovery during sickness [133]. The response of the immune system to infection, with the subsequent release of these immunological mediators, results in the activation of glial cells which consequently lose their modulatory role in the sleep homeostasis [134]. Moreover, at the level of mental disorders, there is a priori reason to expect that at least a substantial proportion of patients with obsessive–compulsive disorder (OCD) would experience a worsening of their disturbances due to the pandemic, with contamination/washing symptoms being the most susceptible [135]. Indeed, stressful life events may precipitate or predispose individuals to the development of OCD symptoms. The intense focus on the potential danger of contamination, as well as the COVID 19 infection, may induce the onset of OCD manifestations in vulnerable subjects, even after months of healing from the disease [136]. Currently, except for epidemiological findings, there are no studies specifically aimed at establishing whether and how COVID-19 infection itself could lead to de novo OCD symptoms or exacerbation of symptoms in people with OCD. In this regard, it is important to take into account the stressful effects of the pandemic, but also it is crucial to consider that infective and/or inflammatory processes have been implicated in some cases of OCD-like symptoms [137], with the evidence of a glial activation occurring in the neurocircuits of OCD [138]. Similarly, de novo appearance of post-traumatic stress disorder (PTSD) spectrum symptoms or their worsening in people who experienced COVID 19 infection is not surprising since the links between inflammation, immune system alterations, and stress-related diseases have been widely demonstrated [139].

Therefore, the risk of increased prevalence of PTSD has also been observed in previous coronavirus pandemics, making its occurrence during this COVID-19 pandemic highly explainable. Some severe cases of COVID-19 result in mortality. The fear of death might be among the many reasons responsible for PTSD amongst these patients. It has been demonstrated that 16% of the severe COVID-19 patients globally had PTSD [140].

A meta-analysis of the survivors among emergency-admitted patients with SARS and MERS infection has revealed that about 39% of them had suffered from PTSD. A history of psychiatric disorders, especially anxiety and depressive disorders, was found as a risk factor for PTSD in intensive care unit survivors. Twelve months after infection, psychiatric symptoms among COVID-19 recovered survivors were reported as 18.3% for depression, 17.9% for PTSD, 16.2% for anxiety, and 13.5% for sleep disturbance [120].

**Fig. 1:**
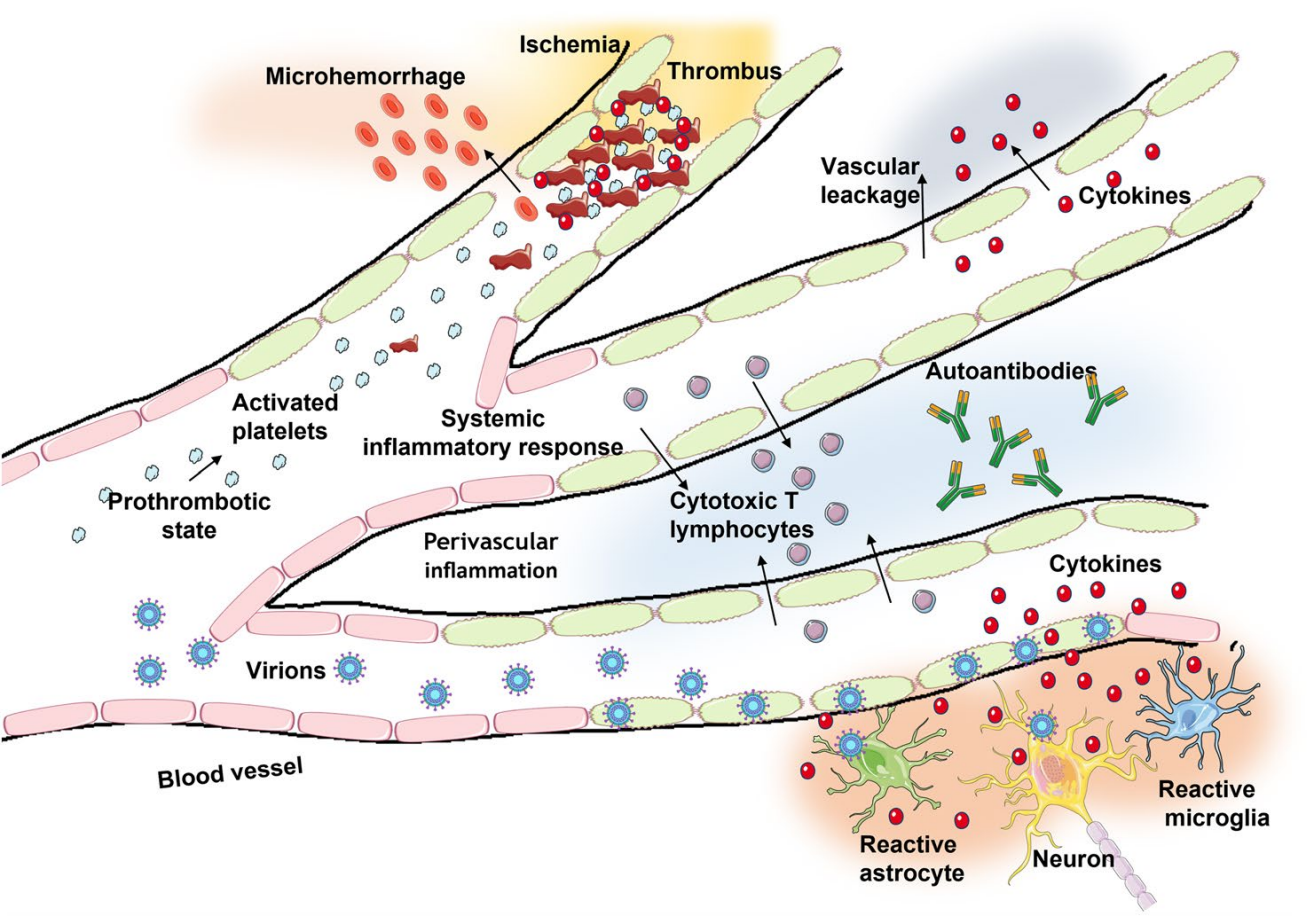
Putative mechanisms implicated in the neurological manifestations of COVID-19

Based on the above reported findings it is possible to state that psychiatric involvement is not uncommon and can lead to severe problems if not detected and managed early. It is recommended that clinicians should be vigilant regarding psychiatric involvement in post COVID-19 patients. Neuroinflammation, blood–brain barrier disruption, thrombotic events, peripheral immune cell invasion into the CNS, glial activation, brain homeostasis impairment, all represent interaction pathways between immune systems and psychopathological mechanisms underpinning such disorders (Fig. 1). In support of this, a recent analysis of brain images taken before and after infection with SARS-CoV-2 demonstrated that even mild COVID-19 is associated with brain structure alterations and brain functioning impairments, suggesting that the effects of SARS-CoV-2 on the CNS need to be very seriously considered [141].

Although evidence suggests that the virus can enter the brain, it seems, however, to predominantly infect vascular and immune cells. In response to immune over activation (and/or virus direct invasion) astrocytes and microglia become reactive. Also, hemorrhages, microvascular infarcts, and thrombotic events are probably critical in the development of the neurological manifestations of SARS-CoV-2 infection.

## Conclusions

COVID-19, initially regarded as specific lung disease, impacts many other organs, affecting their function. Several underlying medical conditions that increase the risk of severe COVID-19 have been identified [142]. Evidence accumulated in the past two years indicates that the brain functions and structure are also damaged by the virus. SARS-CoV-2 infection can indeed cause confusion, memory loss, strokes, psychosis, seizures, and other neurological manifestations. There is also evidence of brain-related abnormalities in COVID-19 patients [141] that may explain the neurological manifestations observed. A study revealed that neurological symptoms appeared in 80% of the people hospitalized with COVID-19 who were surveyed [143]. This depicts a dramatic scenario.

How COVID-19 injuries the brain is becoming clearer. New evidence indicates that the SARS-CoV-2 assault on the brain could be multipronged. The coronavirus might target specific brain cells directly, reduce cerebral blood flow, or trigger the production of immune molecules that can harm brain cells. Astrocytes, tanycytes, infiltrating immune cells, and autoantibodies are probably not the only players in the brain response to the coronavirus leading to the observed neuropsychiatric consequences. Researchers are trying to understand how many brain cells (and what kind of cells) need to be either infected or damaged to cause neurological symptoms. Unfortunately, there isn’t a simple answer. Cerebral cells, including neurons, in some regions of the brain will cause more dysfunction than others, if damaged. This opens a new and never explored field of research. Lastly, whether cerebral effects can be partially reversed, or whether these effects will persist in the long term, remains to be investigated.

## Data Availability

Enquiries about data availability should be directed to the authors.
